# Spontaneous perforation of Meckel diverticulum: A case report

**DOI:** 10.1097/MD.0000000000009506

**Published:** 2017-12-29

**Authors:** Kuan-Ting Liu, Yen-Hung Wu

**Affiliations:** a Department of Emergency Medicine, Kaohsiung Medical University Hospital; b School of Medicine, College of Medicine, Kaohsiung Medical University, Kaohsiung, Taiwan.

**Keywords:** Meckel diverticulum, perforation

## Abstract

**Rationale:**

Meckel diverticulum (MD) is the most common congenital abnormality of the gastrointestinal tract. It is asymptomatic in the majority of patients. Perforation is a rare complication of MD and can be life-threatening.

**Patient concerns:**

A 20-year-old male patient denying previous systemic disease and complaining of epigastric pain for 5 days came to our emergency department for help. Physical examination showed right lower quadrant tenderness without muscle guarding and rebounding pain.

**Diagnosis:**

Blood examination including white blood cell, C reactive protein, liver, and renal function all showed within normal range. Abdominal computed tomography showed suspect MD in the distal ileum and enteritis at the ileum.

**Intervention:**

Perforation of MD was found while in surgery, and Meckel diverticulectomy was performed.

**Outcomes:**

The patient was discharged 7 days after the operation with stable condition.

**Lessons:**

Perforation is an uncommon complication of MD, and the symptom can mimic other acute abdominal conditions such as acute appendicitis while in the emergency space. We should take diagnosis under consideration as a differential diagnosis when we encounter patients whose impression was firstly acute appendicitis.

## 1 Introduction

Perforation is a rare complication of Meckel diverticulum (MD) and can be life-threatening. MD perforation can mimic acute appendicitis. Here, we report a case of MD perforation with initial impression of acute appendicitis. After we contacted the regulations of institutional review board of the Kaohsiung Medical University Hospital, there was no need for ethical approval for this case report article. Informed consent was obtained from the patient.

## 2 Case present

**Figure 1:**
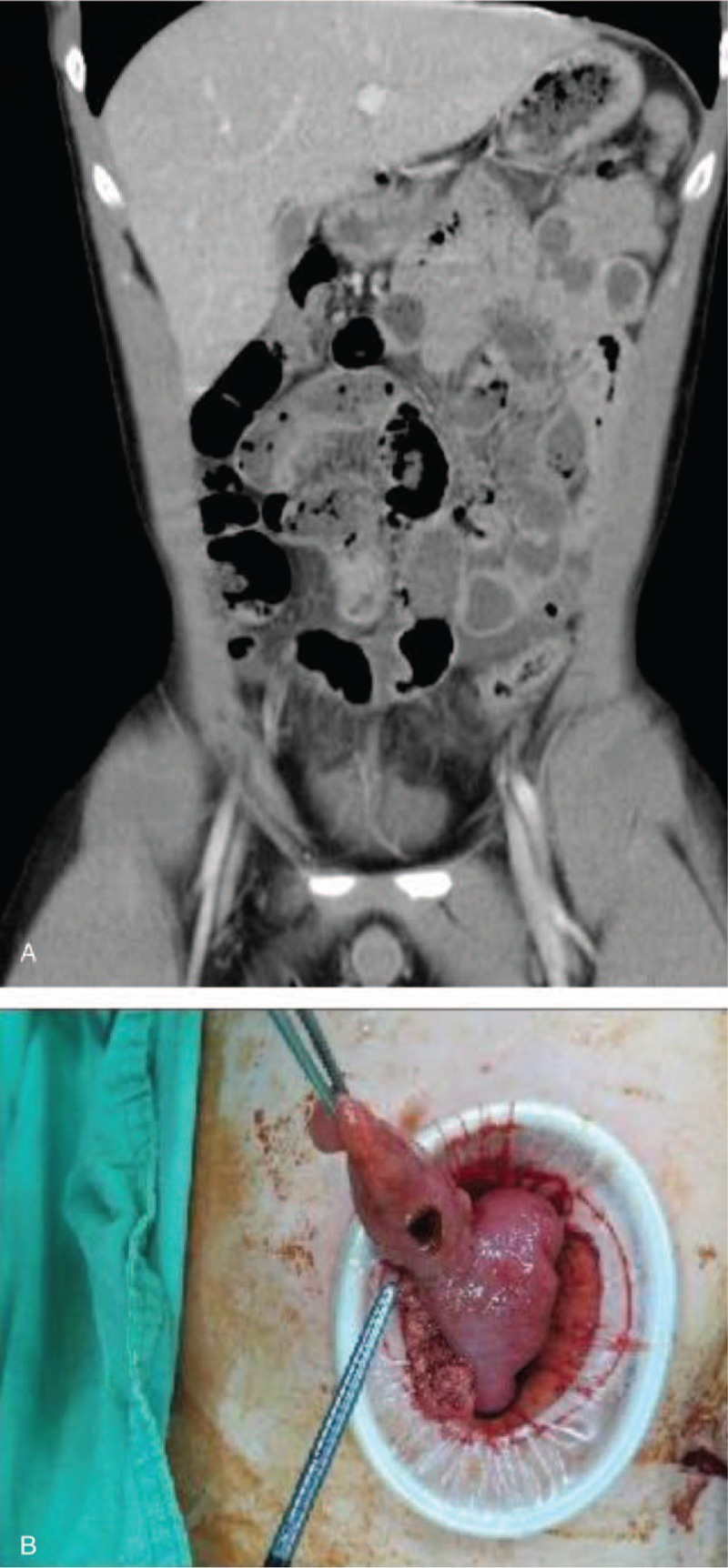
(A) Computed tomography showed suspect Meckel diverticulum in the distal ileum, (B) Meckel diverticulum with perforation, about 0.6 cm in diameter.

A 20-year-old Taiwanese male patient denying previous systemic disease and complaining of epigastric pain for 5 days came to our emergency department for help. The status on arrival showed as below: body temperature 36.7 °C, blood pressure 137/85 mm Hg, pulse rate 100 beats per minute, with clear consciousness. According to the patient, he also had nausea and poor appetite condition, but denied fever, chills, diarrhea, constipation, trauma, or chest pain. The pain was dull and progressed after meals, and included back pain sometimes. The physical examination showed right lower quadrant tenderness without muscle guarding and rebounding pain. Then, we prescribed blood examination (include complete blood count, renal function, liver function, electrolyte such as sodium and potassium, coagulation profile, and C reactive protein) and abdominal computed tomography with contrast for the patient with the impression of suspect intraabdominal infection such as acute appendicitis or diverticulitis. The blood examination showed no leukocytosis or C reactive protein elevation, and the liver and renal function both were within normal range. Computed tomography showed suspect MD in the distal ileum (Fig. 1A) and enteritis at the ileum. A surgeon was consulted and operation was suggested. The laparoscope showed severe adhesion with fecal material in the abdominal cavity, then dirty ascites with food and stool material as a combined 250 mL was drained out. MD with perforation, about 0.6 cm in diameter (Fig. 1B), 60 cm from the terminal ileum was noted. Normal appendix was found. Then, Meckel diverticulectomy was performed. After the operation, the patient was admitted to the ward for further treatment and management. Peripheral nutrition and intravenous ertapenem 1 g daily as antibiotic were prescribed for him. The patient was discharged 7 days after the operation with stable condition. The pathology of the specimen was consistent with MD.

## 3 Discussion

MD is the most common congenital abnormality of the gastrointestinal tract. In the majority of patients, it is asymptomatic and usually found within 40 to 100 cm of the ileocecal valve.^[1]^ In this case, the lesion was found at 60 cm from the terminal ileum. MD is reported to occur in 2% of the population.^[2]^

Most patients with MD are asymptomatic; the total lifetime complication rate has been reported to be around 4%.^[3]^ The complications of MD include common complications such as hemorrhage, bowel obstruction, and diverticulitis; uncommon complications include conditions such as enterolith formation, perforation, and neoplasm. The perforation of MD can mimic acute appendicitis as acute abdomen.^[4,5]^ The perforation of MD is usually secondary to diverticulitis, gangrene, and peptic ulcer,^[6]^ but it can also be induced by trauma^[7]^ and foreign body.^[8,9]^ Symptomatic MD such as gastrointestinal bleeding, Meckel diverticulitis, perforation, and bowel obstruction can all be treated by resection of symptomatic MD.

## 4 Conclusion

Perforation is an uncommon complication of MD, and the symptom can mimic other acute abdominal conditions such as acute appendicitis while in the emergency department. We should take diagnosis under consideration as a differential diagnosis when we encounter patients whose impression was firstly acute appendicitis.
